# Residential inequalities in health-related quality of life among women of reproductive age in four regions of Ethiopia: a decomposition analysis

**DOI:** 10.1186/s12905-023-02465-2

**Published:** 2023-06-19

**Authors:** Tigist Shumet, Nigatu Regassa Geda

**Affiliations:** 1 Center for Population Studies, College of Development Studies, Addis Ababa University, Sidist Kilo Campus, PO Box 1176, Addis Ababa, Ethiopia; 2 Health System and Reproductive Health Research Directorate, Ethiopian Public Health Institute, PO Box 1242or 5645, Addis Ababa, Ethiopia; 3 College Pharmacy and Nutrition, University of Saskatchewan, Saskatoon, Canada

**Keywords:** Inequalities, Health related quality of life, Decomposition, Ethiopia

## Abstract

**Background:**

Ethiopian rural-urban disparities in key domains of health-related quality of life among women in reproductive age have been huge. However, sources of such inequalities were not studied well. Therefore, this study aimed to assess inequalities in health-related quality of life among women residing in urban and rural areas in four regions of Ethiopia.

**Methods:**

This study used data extracted from the 2016 Ethiopian Demographic and Health Survey; collected at national level from January 18, 2016, to June 27, 2016. Stratified two stage cluster sampling method were used. The data collected from 2385 women in the age group 15–49 years who were living in four regions (Afar, Benishangul-Gumuz, Gambela, and Somali regions) of Ethiopia were used for this study. The outcome variable, Health-Related Quality of Life (HRQoL), was generated by Principal Component Analysis. Further, Multivariable Ordinary Least Square and Oaxaca decomposition threefold (interaction) were used in the analysis with a *p*-value less than 0.05 and 95% confidence interval to declare statistical significances.

**Results:**

Women education, region, religion, wealth index, and husband/partner education were identified as predictors of Health-Related Quality of Life. Women residing in rural areas had far lower health-related quality of life than those living in urban areas. The wealth index and educational level of women were the largest contributor of the inequality in health-related quality of life.

**Conclusion:**

A substantial inequality in quality of life exist between women who reside in rural and urban areas in those four regions of Ethiopia. The socioeconomic factors more importantly wealth index and educational attainment explained the significant portion of the reported rural-urban disparities. Therefore, Policymakers and local administrators should pay more attention on interventions that promote education and narrowing gap in wealth in rural and urban settings.

## Background

Health-Related Quality of Life (HRQoL) is referring to how well a person performs in his/her life and his or her realized well-being in terms of physical, mental, and social domains of health [1, 2]. Health-Related Quality of Life also consists of whether an individual can perform a range of activities of day-to-day life such as bathing or dressing him- or herself which is related to the physical functioning domain of health [1]. There are many ideas and notes about what HRQoL is and how it is being measured. A measure of HRQoL was the degree to which people’s contentment requirements are satisfied [3]. Health-Related Quality of Life is also measured with mortality, morbidity, service utilization, and subjective reports of illness [4].

An increasing concern is growing on the disparities observed in health-related quality of life of different population groups (Example: poor vs. rich and rural vs. urban). The leading causes of health inequalities are unequal distribution of income, power, and wealth among the population. Health inequalities within states are primarily associated with the gradient in social factors that can influence, for example, disease onset and response to treatment [5, 6].

In the context of Ethiopia, inequality in maternal health and other socioeconomic dimensions are extremely high. For instance, even though the use of maternal health services had improved in Ethiopia, most of the mothers do not attend the recommended minimum number of four antenatal care (ANC) visits by WHO [7–9]. The Ethiopian Demographic and Health Surveys (EDHS) conducted from 2000 to 2016 indicated there were significant rural-urban difference in delivery care [8]. There are huge disparities in contraceptive uses and choices in Ethiopia, where there is unacceptably low contraceptive prevalence rate (CPR) in Somali and Afar regions. This is further corroborated by very low demand for family planning (FP) in those two regions (i.e. less than 30%) [8].

The prevalence of under nutrition among women in a reproductive age remains high in Ethiopia. Both the 2011 and 2016 EDHS reported close to 30% (i.e., more than one in four) rate of under nutrition with body mass index (BMI) < 18.5 for women. The country is battling against high rates of both macro and micronutrient deficiencies. The most common forms of malnutrition in Ethiopia include acute and chronic under nutrition, vitamin A deficiency (VAD), iron deficiency anemia (IDA), and iodine deficiency disorder (IDD) [10]. The rural-urban differences in nutrition-related indicators also remained huge during the last two decades [8]. Getting access to adequate nutrition in rural Ethiopia is a continued challenge for women. Even when food is available, women are more vulnerable to the risk of being malnourished because of their gender roles in their families with multiple responsibilities they have to carry out including but not limited to childbearing, raising kids, taking care of household chores, working on the farm ,and further they also carrying out many other social responsibilities that would add significant work burden and that increase their chance of being malnourished [11].

In Ethiopia, the prevalence of Intimate partner violence (IPV) which is considered an important indicator of women’s health status is unacceptably high. According to 2016 Ethiopian Demographic and Health Survey (EDHS) report, nearly one-quarter (23%) of women had at some point in their lives experienced physical violence. While 10% of the women experienced sexual violence. 34% of married women have experienced spousal violence, whether physical, sexual, or emotional, with emotional violence being the most common [8].

In Ethiopia, despite the marked improvement in health-related quality of life of people at the national level, substantial inequalities among the different socio-economic subgroups persist. Studying the health-related quality of life in different populations i.e. rural and urban in this case would help to identify subgroups with poor health related quality of life, and this, in turn guides policy makers and other actors to improve the existing policies or interventions to enhance the population health status and make a fair distribution of the available resource to leverage the health of the society at large. To the best of the authors’ knowledge, no study has been conducted yet on the four regions of this study focusing on inequalities in HRQoL among women in the reproductive age using a combined indicators as an outcome variable. But previous studies have considered a single measure (such as access to health services, income, employment, etc…) as an outcome variable. Further, those studies were conducted at the local level on a limited sample of respondents drawn from a specific region or district within Ethiopia. The present study hypothesizes that there are substantial rural-urban inequalities in HRQoL among women in four regions of Ethiopia. The study answers the following two questions: (a) What are the major predictors of the health-related quality of life among women in urban and rural areas of Ethiopia, and (b) What are the prime drivers of rural-urban differentials (i.e. source of inequality) in health-related quality of life in four regions of Ethiopia?

## Methods

### Study setting

The study was conducted in four regions of Ethiopia, namely; Afar, Somali, Benishangul Gumuz, and Gambela regions. These all regions have the following common characteristics: geographical peripheral, economic disadvantaged in terms of access to essential amenities, relatively homogeneous population, and distinctive cultural characteristics [12–15]. According to the Central Statistical Agency (CSA) of Ethiopia’s projection of population size for the year 2020, the overall population of Afar was 1.95 million, Somali was 6.20 million, Benishangul-Gumuz was 1.16 million, and Gambella was 0.478 million. When we see the percentage of populations resides in urban areas in those study regions; Afar has 21%, Somali has 15%, Benishangul-Gumuz has 23.6%, and Gambella has 36.6% [16].

### Data source and the study population

The study used secondary data from the 2016 EDHS. The data were collected at a national level from all nine regional states and two city administrations of Ethiopia from January 18, 2016, to June 27, 2016. But this study used data collected from only those four regions of Ethiopia (i.e., Afar, Benishangul Gumuz, Gambela, and Somali regions ). During data collection, the eligibility criteria to include respondents of the study was *“all women aged 15–49 years, who were either permanent residents of the selected households or visitors who slept in the household the night before the survey’’.* To generate the EDHS data, five different questionnaires were used. 4160 women were interviewed on those four region. Among those, the present study used data collected by the household and woman’s questionnaires from 2385 women who were eligible for all composite indicator of outcome variables (344 from urban and 1990 from rural) in the age group 15–49 years situated in four regions of Ethiopia. In general, EDHS used a Stratified two-stage cluster sampling. In the first stage, 645 Enumeration Areas (EA) were selected by using a probability proportional to EA size. Therefore, a total of 222 EAs were used for the present study i.e., 53EAs from Afar 69 EAs from Somali, 50 EAs from Benishangul Gumuz, and 50 EAs from Gambela, Household listing was performed to have a sample frame for the second stage sampling (i.e., for household selection). In the second stage, a fixed number of 28 households per cluster have been selected with an equal probability using a systematic random sampling technique [8].

### Measures of outcome variables

The main outcome variable of this study was Health-Related Quality of Life (HRQoL) which was generated by using the principal component analysis (PCA). This method sets a factor score that explains the maximum possible variations in the input variables used. The factor score was created from four generic indicators which comprise various variables i.e. physical quality indicators (anemia and BMI), service access indicators (ANC, postnatal care (PNC), contraceptive utilization, health facility delivery, and iron utilization), and household wellbeing indicators (media access, household sanitation, decision making, substance abuse, health insurance, and IPV), and functional indicator (presence of temporary or permanent quit of doing normal activity). The Factor Analysis Score (FAC) which had the highest Eigenvalues (Eigenvalue = 2.8) was taken and was used as a multiplier of all the 14 individual HRQoL indicators considered. The final aggregate indicator was labeled as the HRQoL index which had a right-skewed distribution with a mean of 2.2 and standard deviation (SD) of 31.8. The values of the outcome variable ranged between − 31 and 159.

### Exposure variables

The exposure/ independent variables of this study include woman’s educational level, regions, wealth index, husband/partner education status, religion, sex of the household head, age of household head, total number of children ever born, ethnicity, relationship to household head and marital status.

### Statistical analysis

**Fig. 1:**
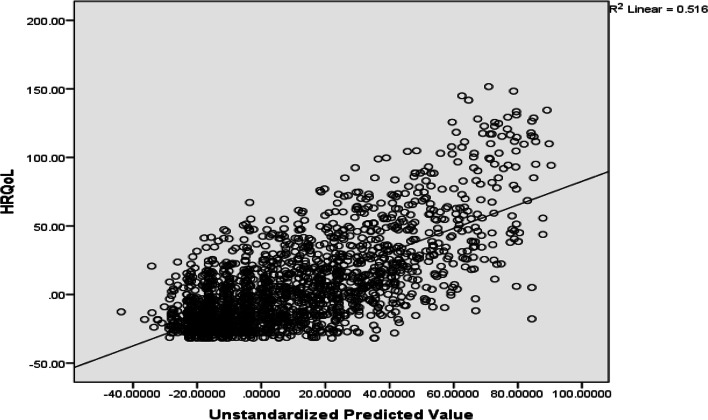
Scatter plot for health related quality of life and predictor variables in urban residence

**Fig. 2:**
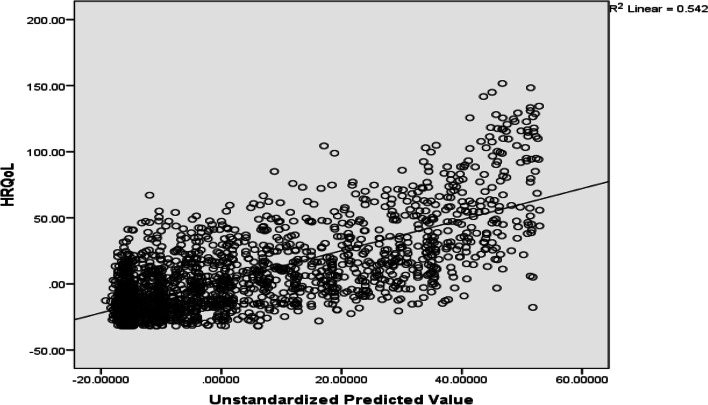
Scatter plot for health related quality of life and predictor variables in rural residence

Data cleaning and analysis were done using SPSS-v20 and STATA -v14. The analysis began with describing the characteristics of respondents using frequency and percentages. Multicollinearity among the explanatory variables was checked using the Variance Inflation Factor (VIF), with VIFs > 2.5 indicating significant multicollinearity problems [17]. The linearity between parameter of independent variable and outcome variable were evaluated by using a scatter plot as shown in Figs. 1 and 2. Linear regression models ordinary Least Square parameter estimation method were used to examine the associations between covariates and the outcome variable [17]. As a rule of thumb, potential variables with a *p*-value < 0.20 were further tested in the multivariable Ordinary Least Square (OLS) regression. The study also used a *p*-value less than 0.05 and 95% confidence interval for declaration of statistical significance. The rural-urban disparities in quality of life among women were estimated using Oaxaca decomposition with the threefold (interaction) decomposition type. The technique is generally used to investigate the group differences in any (continuous and unbounded) outcome variable [18]. The “threefold (reverse)” selection perspective of group 2 indicates that group 1 (i.e., urban adults with a higher average HRQoL) are selected as the reference group for analysis.

## Results

### Characteristics of respondents

Table 1 summarizes the background characteristics of women respondents of the study. Among the total respondents, most of them (48%) were found in the age group 25 to 34 years. In addition, the study also found 70% of the study participants have no education and 61% of them were also categorized in the “poorest” household wealth group.

**Table 1:** Background characteristics of women respondents, EDHS 2016, *n* = 2385

Characteristics	Number	Percent
Women age		
15–24	694	29.1
25–34	1138	47.7
> 34	553	23.2
Women education		
No education	1668	69.9
Primary	502	21.0
Secondary and higher	215	9.0
Working status		
Not working	1717	72.0
Working	668	28.0
Household age		
15–24	251	10.5
25–34	878	36.8
> 34	1256	52.7
Husband education		
No education	1338	59.6
Primary	486	21.7
Secondary and higher	420	18.7
Sex of household head		
Male	1616	67.8
Female	769	32.2
Household size final		
<=3	377	15.8
>=4	2008	84.2
Wealth index combined		
Poorest	1456	61.0
Poorer	276	11.6
Middle	172	7.2
Richer	183	7.7
Richest	298	12.5
Total	2385	100.0

### Rural-urban differentials in the predictors of health-related quality of life

The result in Table 2 presented the bivariate OLS regression. It is noted that, except marital status all the other independent variables of the study had a ***p***-value < 0.2, and hence, all of those with ***p***-value < 0.2 were considered for further analysis in the multiple ordinary least square regression model.

**Table 2:** Bivariate ordinary least square regression for selected predictors of HRQoL in urban and rural Ethiopia

	Urban				Rural			
	Beta	(95%CI)		*p*-value	Beta	(95%CI)		*p*-value
Women Age								
15–24 ^RC^								
25–34	-0.09	-16.56	-2.43	**0.14**	-0.05	-4.72	-0.19	**0.07**
> 34	-0.18	-31.56	-6.83	**0.00**	-0.06	-6.29	-0.57	**0.02**
Household age								
15–24 ^RC^								
25–34	-0.11	-25.90	-5.93	0.22	0.05	-1.15	-6.02	**0.18**
> 34	-0.19	-31.14	-0.20	**0.05**	0.04	-3.23	-3.67	0.90
Household Size								
<=3 ^RC^								
>=4	-0.27	-31.51	-11.78	**0.00**	-0.05	-6.43	-5.60	**0.02**
Women Education								
No education	0.27	16.47	34.73	**0.00**	0.27	13.57	18.50	**0.00**
Primary	0.51	37.36	54.87	**0.00**	0.23	20.11	29.15	**0.00**
Secondary								
Region								
Afar ^RC^								
Somali	0.34	-40.51	-18.17	**0.00**	0.01	-1.99	-3.09	0.67
Benishangul	0.13	2.99	-34.11	**0.00**	0.42	20.17	-25.48	**0.00**
Gambela	0.01	-13.43	-16.24	0.81	0.19	8.47	-14.22	**0.00**
Religion								
Orthodox ^RC^								
Protestant	-0.44	-54.63	-31.26	**0.00**	-0.32	-23.30	-14.91	**0.00**
Muslin	-0.57	-57.20	-36.94	**0.00**	-0.46	-27.35	-19.88	**0.00**
Other	-0.08	-44.84	-4.61	**0.11**	-0.17	-30.39	-16.89	**0.00**
Wealth Index								
Poorest ^RC^						.	.	.
Poorer	0.05	-10.32	27.55	0.37	0.29	18.09	23.18	**0.00**
Middle	0.06	-7.68	-31.74	0.23	0.32	24.96	31.30	**0.00**
Richer	0.12	1.74	33.38	**0.03**	0.41	34.15	40.58	**0.00**
Richest	0.60	42.53	64.63	**0.00**	0.31	46.49	58.36	**0.00**
Household sex								
Male ^RC^								
Female	-0.08	-15.68	-1.74	**0.12**	-0.08	-6.49	-2.04	**0.00**
Husband/Partner education								
No education ^RC^								
Primary	0.33	23.71	46.72	**0.00**	0.27	12.84	17.80	**0.00**
Secondary	0.48	30.49	48.19	**0.00**	0.24	13.40	19.49	**0.00**
Women employment								
Not working ^RC^								
Working	0.30	17.44	34.34	**0.00**	0.22	9.31	13.89	**0.00**
Marital status								
Otherwise ^RC^								
Married	0.13	-12.09	15.70	0.80	-0.01	-5.09	3.52	0.72
Relationship with household head								
Other ^RC^								
Head	0.16	-11.18	40.08	0.27	0.04	-5.56	9.84	0.58
Wife	0.26	-3.50	46.48	**0.09**	0.13	-1.33	13.76	**0.11**
Daughter	0.18	-1.61	55.43	**0.06**	0.02	-6.24	10.93	0.59
Daughter in low	0.02	-43.32	62.08	0.73	0.05	0.08	26.06	**0.05**
Total children ever born								
<=2	-0.33	-37.08	-19.79	**0.00**	-0.09	-7.13	-2.11	**0.00**
3–5	-0.32	-45.51	-23.46	**0.00**	-0.16	10.91	-5.67	**0.00**
>=6 ^RC^								

*RC *Reference category

Table 3 presents the results of a multiple ordinary least square regression. The result revealed; in rural parts of those four regions of the study, the mean HRQoL is higher for women with a primary level of education by 11% compared with the reference no-education category. Similarly, women with secondary and higher education had higher mean HRQoL compared to women with no education (i.e., increased by 11%). The HRQoL is higher for women living in richer and richest households by 29% and 25%, respectively (i.e. β = 0.29 and β = 0.25, respectively) compared to the reference i.e., those living in poorest households in the same locality. When we see the regression output for urban (model 2), it is observed that the mean HRQoL is higher by 10% for those women richer household wealth index (β = 0.10, 99.5% CI: -0.43, 27.41). And higher by 40% for those women with richest household wealth index (β = 0.40, 95% CI: 25.49, 45.58). The mean HRQoL is higher for those women with secondary and higher by 23% compared to women with no education (β = 0.23).

**Table 3:** Multiple ordinary least square regression for selected predictors of HRQoL among women in rural and urban Ethiopia (*n* = 2385)

	Rural				urban			
	Beta	95% CI)		*p*-value	Beta	95% CI)		*p*-value
Women Age								
15–24 ^RC^								
25–34	0.02	-1.92	3.61	0.55	0.08	-2.30	14.90	0.15
> 34	0.03	-2.15	5.40	0.40	0.03	-9.23	16.49	0.58
Household head age								
15–24 ^RC^								
25–34	0.02	-2.30	4.42	0.54	-0.07	0.38	-19.28	7.38
> 34	0.04	-2.07	5.81	0.35	-0.11	0.22	-24.09	5.66
Household Size ^RC^								
<=3	-0.03	-5.08	0.74	0.14	0.02	0.71	-7.34	10.79
>=4								
Women Education								
No education ^RC^								
Primary	0.11	4.21	9.11	**0.00**	0.08	-1.34	17.05	0.09
Secondary and higher	0.11	7.79	16.97	**0.00**	0.23	10.39	31.33	**0.00**
Region								
Afar ^RC^								
Somali	-0.06	-5.40	-0.99	**0.00**	-0.07	0.26	-16.10	4.33
Benishangul	0.15	5.30	11.54	**0.00**	0.08	0.10	-2.21	24.45
Gambela	0.14	4.44	12.80	**0.00**	0.11	0.11	-1.97	20.50
Religion								
Orthodox ^RC^								
Protestant	-0.25	-19.07	-11.20	**0.00**	-0.38	-47.88	-25.38	**0.00**
Muslin	-0.04	-5.83	1.53	0.25	-0.22	-29.21	-7.78	**0.00**
Other	-0.07	-15.14	-3.99	**0.00**	-0.06	-38.87	3.68	**0.11**
Wealth Index								
Poorest ^RC^								
Poorer	0.21	11.94	17.30	**0.00**	0.00	-15.19	16.88	0.92
Middle	0.23	17.22	23.83	**0.00**	0.07	-2.54	31.35	0.09
Richer	0.29	22.55	29.47	**0.00**	0.10	0.43	27.41	**0.04**
Richest	0.25	35.17	46.95	**0.00**	0.40	25.49	45.58	**0.00**
Household sex								
Male ^RC^								
Female	-0.05	-8.48	3.63	0.43	-0.09	0.44	-28.82	12.60
Husband/Partner education								
No education ^RC^								
Primary	0.09	2.81	7.26	**0.00**	0.10	1.36	20.99	**0.03**
Secondary	0.07	2.26	8.30	**0.00**	0.16	3.75	22.15	**0.01**
Women employment								
Not working ^RC^								
Working	0.04	0.31	4.37	**0.02**	0.07	0.11	-1.25	12.83
Relationship with household head								
Other ^RC^								
Head	0.06	-5.04	8.91	0.59	0.22	0.08	-2.13	43.57
Wife	0.00	-5.39	8.22	0.68	0.18	0.21	-8.85	39.87
Daughter	-0.01	-8.80	6.57	0.78	0.19	0.01	9.00	54.72
Daughter in low	0.03	-2.71	19.13	0.14	0.02	0.65	-28.99	46.46
Total children ever born								
<=2 ^RC^								
3–5	-0.04	-4.61	0.85	0.18	-0.13	-20.09	-2.08	**0.02**
>=6	-0.03	-4.94	1.75	0.35	-0.01	-13.66	12.06	0.90
Number of obs = 1900	AIC = 3293	BIC = 3401			Number of obs = 344		AIC = 16,391	BIC =16,547

### Decomposition analysis

Table 4 presented the Oaxaca decomposition with the threefold (interaction) decomposition type. In this study, the “threefold (reverse)” selection perspective of group 2 indicates that group 1 (i.e., urban adults with a higher average HRQoL) are selected as the reference for the analysis.

**Table 4:** Blinder-Oaxaca decomposition for selected risk factors of women health-related quality of life for rural urban Ethiopia

Group 1: Urban Group 2: Rural						
	Coef.	Std.Err.	z	P > z	95% CI	
Overall						
Group_1	40.956	2.241	18.280	0.000	36.564	45.348
Group_2	-4.829	0.544	-8.880	0.000	-5.895	-3.764
Difference	45.785	2.306	19.860	0.000	41.266	50.305
Endowments	34.778	1.591	21.860	0.000	31.660	37.896
Coefficients	4.145	3.573	1.160	0.246	-2.857	11.148
Interaction	6.862	3.420	2.010	0.045	0.160	13.564
Endowments						
Women age	-0.126	0.100	-1.260	0.207	-0.322	0.070
Household age	-0.001	0.006	-0.090	0.931	-0.013	0.012
Women education	2.922	0.620	4.720	0.000	1.708	4.137
Household size	0.207	0.124	1.670	0.096	-0.037	0.450
Region	-0.130	0.178	-0.730	0.466	-0.478	0.219
Sex of household	-0.047	0.057	-0.830	0.407	-0.159	0.064
Religion	0.342	0.221	1.550	0.121	-0.091	0.775
Household wealth index	28.920	1.474	19.620	0.000	26.031	31.809
Husband education	1.904	0.518	3.680	0.000	0.890	2.919
Employment status	0.071	0.106	0.670	0.505	-0.138	0.280
Marital status	0.372	0.159	2.340	0.019	0.061	0.683
Relationship to the household	-0.003	0.018	-0.200	0.844	-0.038	0.031
Total children ever born	0.346	0.261	1.330	0.184	-0.165	0.857
Coefficients						
Women age	9.044	6.625	1.370	0.172	-3.941	22.029
Household age	-11.730	8.299	-1.410	0.158	-27.996	4.536
Women education	1.731	0.877	1.980	0.048	0.013	3.449
Household size	-0.705	9.107	-0.080	0.938	-18.554	17.145
Region	-3.490	4.745	-0.740	0.462	-12.790	5.810
Sex of household	-3.798	5.642	-0.670	0.501	-14.856	7.261
Religion	-23.804	6.418	-3.710	0.000	-36.383	-11.225
Household wealth index	-0.328	2.097	-0.160	0.876	-4.438	3.782
Husband/ partner education	1.087	1.217	0.890	0.372	-1.298	3.472
Employment status	-4.317	11.906	-0.360	0.717	-27.653	19.019
Marital status	5.903	4.836	1.220	0.222	-3.575	15.380
Relationship to the household	10.528	6.467	1.630	0.104	-2.148	23.203
Total children ever born	-2.110	3.403	-0.620	0.535	-8.781	4.561
_cons	26.134	20.761	1.260	0.208	-14.557	66.825
Interaction						
Women age	-0.354	0.320	-1.110	0.269	-0.982	0.273
Household age	-0.020	0.183	-0.110	0.912	-0.378	0.338
Women education	3.355	1.715	1.960	0.050	-0.007	6.717
Household size	0.028	0.365	0.080	0.938	-0.687	0.743
Region	-0.485	0.665	-0.730	0.466	-1.788	0.818
Sex of household	-0.104	0.174	-0.600	0.551	-0.444	0.237
Religion	2.531	0.798	3.170	0.002	0.967	4.095
Household wealth index	-0.533	3.403	-0.160	0.876	-7.202	6.136
Husband education	1.488	1.669	0.890	0.373	-1.783	4.759
Employment status	0.084	0.235	0.360	0.721	-0.376	0.544
Marital status	0.339	0.306	1.110	0.268	-0.260	0.939
Relationship to the household	-0.088	0.224	-0.390	0.696	-0.526	0.351
Total children ever born	0.620	1.003	0.620	0.537	-1.347	2.586

In this study, the mean predicted HRQoL among women was 40.956 for women residing in urban (group_1) and − 4.829 for women residing in rural (group_2) Ethiopia, which yields a HRQoL disparity of 45.785. In general, about 507% (34.778/6.862) of the disparity was due to the difference in the distribution of the predictors (endowments). Among them, the wealth index (28.92/6.862 = 421%) contributed a substantial portion of the disparities. The differential effect of women age (9.044/6.862 = 132%) had the greatest contribution and was attributed to the differential effect of the covariate entered in the model (coefficients effect) including the general effect of unknown factors (_cons).

As shown in Table 4, the differences in the level of observed covariates (the explained component) accounted for about 90.94% (41.64/45.785) of the total disparity.

## Discussion

The present study assessed the inequality in health-related quality of life among women in urban and rural settings of four regions of Ethiopia using 2016 EDHS data. Among all predictors, wealth index, educational level of women and their husbands (partners) had a substantial contribution to the existing rural-urban differentials of inequality in the quality of life of women.

The study found that there was a significant variation in the quality of life among women residing in urban and rural areas in the four regions of Ethiopia; It was found that women residing in rural areas had far lower HRQoL than those women residing in urban areas. This finding is consistent with previous studies conducted around the world [19–21].

In the decomposition analysis, the household wealth index, educational level of women, and their husband (partner) were found to explain the inequality in health-related quality of life among women in both urban and rural Ethiopia. This study revealed both of those explaining factors i.e., household wealth index and the educational level (socio-economic variables) of those women were contributing to broadening the gap in health-related quality of life among women in rural-urban areas of Ethiopia. This finding is consistent with a study done by Abosse and colleagues (2021) who found significant urban-rural inequalities in maternal healthcare utilization in selected countries in Sub-Saharan Africa which included Ethiopia [7]. The finding of this study also coincides with the study done by other scholars which highlight the need to minimize the wealth imbalance and to improve the low levels of education between mothers to enhance the usage of maternal health service in sub-Saharan African countries [22]. Furthermore, previous studies also indicated that education is a fundamental determinant of QOL of individuals i.e., individuals who have limited educational preparation (skills and competencies) may be barred from securing good jobs and have fewer outlooks for economic prosperity; in addition, those individuals who are early schools’ leavers encounter higher risk of social exclusion, poverty and usually may have a very little opportunity in participation for voting and political affairs. Since, education usually increases individuals perception of the world they live in, and their ability how to influence others [23, 24].

Even though the EDHS-type surveys used advance survey methodology and large sample size used for the present analysis, the cross-sectional nature of the data limits the possibilities of establishing cause-effect relationship between the factors and the outcome of interest. As EDHS surveys are primarily designed to track trends in population, health, and nutritional programs in Ethiopia, they lack some relevant variables for the construction of the HRQoL index. In addition, there may be data quality differences from respondents drawn from rural and urban areas. Despite these limitations, this study can be taken as a springboard for further analysis on the subject i.e., inequality in the health related quality of life. In addition, the findings can be useful at both national as well as a regional levels as input for formulation of strategies for reducing the inequalities in HRQoL among women who reside in rural and urban areas.

## Conclusion

The study found a substantial inequality in the quality of life among women who reside in rural and urban areas in the four concerned regions of Ethiopia. The finding further witnessed that two socioeconomic factors (namely, wealth index and educational attainment) explained a larger proportion of the inequality in the quality of life of women in urban/ rural areas in the four regions of Ethiopia. The findings imply that policymakers and local administrators should pay more attention to interventions that promote education and narrow down the gap in wealth status of households.

## Data Availability

The data used for this manuscript is a secondary data which is available online in ICF international/DHS program (https://dhsprogram.com/data/new-user-registration.cfm). The data can be accessed with submission of a request and the approval of ICF international/DHS program.
